# Triaqua­dichlorido[5-(4-pyridinio)tetra­zolato-κ*N* ^2^]cobalt(II) monohydrate

**DOI:** 10.1107/S1600536809024337

**Published:** 2009-07-01

**Authors:** Bo Wang

**Affiliations:** a Ordered Matter Science Research Center, College of Chemistry and Chemical Engineering, Southeast University, Nanjing 210096, People’s Republic of China

## Abstract

The title compound, [CoCl_2_(C_6_H_5_N_5_)(H_2_O)_3_]·H_2_O, was synthesized by hydro­thermal reaction of CoCl_2_ with 4-(2*H*-tetra­zol-5-yl)pyridine. The Co^II^ cation is coordinated by two Cl^−^ ions, one N atom from the 5-(4-pyridinio)tetra­zolate zwitterion and three O atoms from three water mol­ecules in a distorted octa­hedral geometry. In the crystal, mol­ecules are linked into a three-dimensional network by N—H⋯Cl hydrogen bonds and O—H⋯O/N/Cl hydrogen bonds involv­ing both coordinated and uncoordinated water mol­ecules. Strong π–π stacking is present between parallel pyridinium and tetra­zolate rings [centroid–centroid distances = 3.411 (2) and 3.436 (2) Å].

## Related literature

For general background to the chemistry of tetra­zole derivatives, see: Fu *et al.* (2007 ▶, 2008 ▶); Huang *et al.* (1999 ▶); Liu *et al.* (1999 ▶); Wang *et al.* (2005 ▶). For the crystal structures of related compounds, see: Dai & Fu (2008 ▶); Wen (2008 ▶); Wittenberger & Donner (1993 ▶).
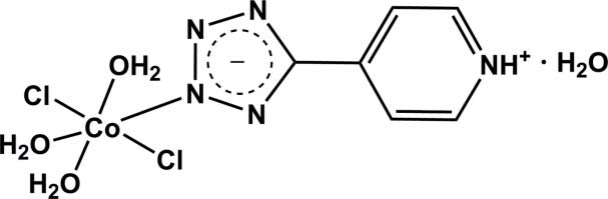


## Experimental

### 

#### Crystal data

[CoCl_2_(C_6_H_5_N_5_)(H_2_O)_3_]·H_2_O
*M* *_r_* = 349.04
Triclinic, 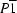

*a* = 6.4900 (13) Å
*b* = 9.842 (2) Å
*c* = 11.159 (2) Å
α = 110.72 (3)°
β = 97.05 (3)°
γ = 106.27 (3)°
*V* = 620.1 (3) Å^3^
*Z* = 2
Mo *K*α radiation
μ = 1.83 mm^−1^
*T* = 298 K
0.15 × 0.15 × 0.10 mm

#### Data collection

Rigaku Mercury2 diffractometer
Absorption correction: multi-scan (*CrystalClear*; Rigaku, 2005 ▶) *T*_min_ = 0.762, *T*_max_ = 0.841
6551 measured reflections
2842 independent reflections
2619 reflections with *I* > 2σ(*I*)
*R*_int_ = 0.019

#### Refinement

*R*[*F* ^2^ > 2σ(*F* ^2^)] = 0.026
*wR*(*F* ^2^) = 0.080
*S* = 1.18
2842 reflections
163 parameters
H-atom parameters constrained
Δρ_max_ = 0.34 e Å^−3^
Δρ_min_ = −0.49 e Å^−3^

### 

Data collection: *CrystalClear* (Rigaku, 2005 ▶); cell refinement: *CrystalClear*; data reduction: *CrystalClear*; program(s) used to solve structure: *SHELXS97* (Sheldrick, 2008 ▶); program(s) used to refine structure: *SHELXL97* (Sheldrick, 2008 ▶); molecular graphics: *SHELXTL* (Sheldrick, 2008 ▶); software used to prepare material for publication: *SHELXTL*.

## Supplementary Material

Crystal structure: contains datablocks I, global. DOI: 10.1107/S1600536809024337/ci2831sup1.cif

Structure factors: contains datablocks I. DOI: 10.1107/S1600536809024337/ci2831Isup2.hkl

Additional supplementary materials:  crystallographic information; 3D view; checkCIF report

## Figures and Tables

**Table 1:** Hydrogen-bond geometry (Å, °)

*D*—H⋯*A*	*D*—H	H⋯*A*	*D*⋯*A*	*D*—H⋯*A*
O3*W*—H3*WA*⋯O4*W*^i^	0.95	1.92	2.858 (3)	173
O3*W*—H3*WB*⋯O4*W*^ii^	0.86	1.91	2.761 (2)	170
O1*W*—H1*WB*⋯N4^iii^	0.88	2.01	2.848 (2)	157
O2*W*—H2*WA*⋯N2^iv^	0.83	2.24	2.999 (2)	153
O4*W*—H4*WA*⋯N5^v^	0.83	2.11	2.935 (3)	173
N1—H1*A*⋯Cl2^v^	0.86	2.41	3.180 (2)	149
O1*W*—H1*WA*⋯Cl2^vi^	0.94	2.32	3.254 (2)	174
O2*W*—H2*WB*⋯Cl1^vii^	0.91	2.42	3.300 (2)	163
O4*W*—H4*WB*⋯Cl1^iv^	0.88	2.38	3.249 (2)	168

Symmetry codes: (i) 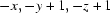; (ii) 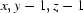; (iii) 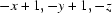; (iv) 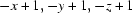; (v) 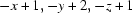; (vi) 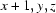; (vii) 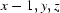.

**Fig. 1.**
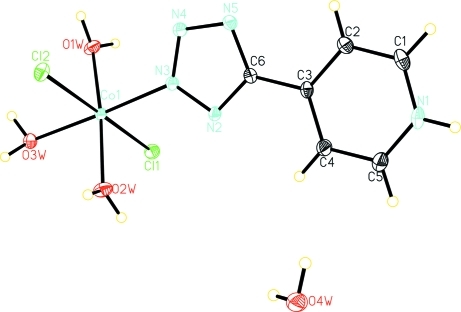
The molecular structure of the title compound, with the atomic numbering scheme. Displacement ellipsoids are drawn at the 30% probability level.

**Fig. 2.**
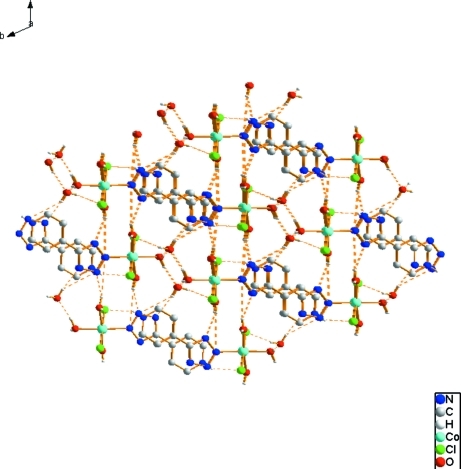
The crystal packing of the title compound, viewed along the a axis, showing the three dimensionnal hydrogen-bonded network. H atoms not involved in hydrogen bonding (dashed line) have been omitted for clarity.

## supplementary crystallographic information

### Comment

The tetrazole functional group has found a wide range of applications in coordination chemistry as a ligand, in medicinal chemistry as a metabolically stable surrogate for the carboxylic acid group, and in materials science as a high density energy materials, dielectric and luminescence materials (Wang *et al.*, 2005; Fu *et al.*, 2008; Fu *et al.*, 2007; Huang *et al.*, 1999; Liu *et al.*, 1999; Wittenberger *et al.*,1993). We report here the crystal structure of the title compound, triaqua-dichloro-[4-(2*H*-tetrazol)pyridinum]cobalt(II) monohydrate.

The Co^II^ cation is coordinated by two Cl^-^ ions, one N atom from the pyridinio-4-(2H-tetrazolate) zwitterion and three O atoms from three water molecules in a distorted octahedral geometry. The pyridine N atom of the organic ligand is protonated. The pyridinium and tetrazolate rings are almost coplanar, with a dihedral angle of 3.7 (1)°. The geometric parameters of the tetrazolate ring are comparable to those in related molecules (Wittenberger *et al.*, 1993; Dai & Fu 2008; Wen 2008).

The molecules are linked into a three-dimensional network by intermolecular O—H···O, O—H···N, N—H···Cl and O—H···Cl hydrogen bonds (Table 1 and Fig.2).

### Experimental

A mixture of 4-(2*H*-tetrazol-5-yl)pyridine (0.2 mmol), CoCl_2_ (0.4 mmol), distilled water (1 ml) and a few drops of HCl (6 mol/L) was sealed in a glass tube and maintained at 333 K. Pink block-shaped crystals suitable for X-ray analysis were obtained after 3 d.

### Refinement

H atoms of water molecules were located in difference Fourier maps and in the final stages of refinement they were treated as riding on the parent O atom with *U*_iso_(H) = 1.5*U*_eq_(O). The remaining H atoms were positioned geometrically and treated as riding, with C-H = 0.93 Å, N-H = 0.86 Å, and with *U*_iso_(H) = 1.2*U*_eq_(C,N).

### Crystal data

**Table:**

[CoCl_2_(C_6_H_5_N_5_)(H_2_O)_3_]·H_2_O	*Z* = 2
*M**_r_* = 349.04	*F*(000) = 354
Triclinic, *P*1	*D*_x_ = 1.869 Mg m^−^^3^
Hall symbol: -P 1	Mo *K*α radiation, λ = 0.71073 Å
*a* = 6.4900 (13) Å	Cell parameters from 2619 reflections
*b* = 9.842 (2) Å	θ = 3.4–27.5°
*c* = 11.159 (2) Å	µ = 1.83 mm^−^^1^
α = 110.72 (3)°	*T* = 298 K
β = 97.05 (3)°	Block, pink
γ = 106.27 (3)°	0.15 × 0.15 × 0.10 mm
*V* = 620.1 (3) Å^3^	

### Data collection

**Table:**

Rigaku Mercury2 diffractometer	2842 independent reflections
Radiation source: fine-focus sealed tube	2619 reflections with *I* > 2σ(*I*)
graphite	*R*_int_ = 0.019
Detector resolution: 13.6612 pixels mm^-1^	θ_max_ = 27.5°, θ_min_ = 3.4°
CCD profile fitting scans	*h* = −8→8
Absorption correction: multi-scan (*CrystalClear*; Rigaku, 2005)	*k* = −12→12
*T*_min_ = 0.762, *T*_max_ = 0.841	*l* = −14→14
6551 measured reflections	

### Refinement

**Table:**

Refinement on *F*^2^	Primary atom site location: structure-invariant direct methods
Least-squares matrix: full	Secondary atom site location: difference Fourier map
*R*[*F*^2^ > 2σ(*F*^2^)] = 0.026	Hydrogen site location: inferred from neighbouring sites
*wR*(*F*^2^) = 0.080	H-atom parameters constrained
*S* = 1.18	*w* = 1/[σ^2^(*F*_o_^2^) + (0.0344*P*)^2^ + 0.391*P*] where *P* = (*F*_o_^2^ + 2*F*_c_^2^)/3
2842 reflections	(Δ/σ)_max_ = 0.001
163 parameters	Δρ_max_ = 0.34 e Å^−^^3^
0 restraints	Δρ_min_ = −0.49 e Å^−^^3^

### Special details

**Table:**

Geometry. All esds (except the esd in the dihedral angle between two l.s. planes) are estimated using the full covariance matrix. The cell esds are taken into account individually in the estimation of esds in distances, angles and torsion angles; correlations between esds in cell parameters are only used when they are defined by crystal symmetry. An approximate (isotropic) treatment of cell esds is used for estimating esds involving l.s. planes.
Refinement. Refinement of F^2^ against ALL reflections. The weighted R-factor wR and goodness of fit S are based on F^2^, conventional R-factors R are based on F, with F set to zero for negative F^2^. The threshold expression of F^2^ > 2sigma(F^2^) is used only for calculating R-factors(gt) etc. and is not relevant to the choice of reflections for refinement. R-factors based on F^2^ are statistically about twice as large as those based on F, and R- factors based on ALL data will be even larger.

### Fractional atomic coordinates and isotropic or equivalent isotropic displacement parameters (Å^2^)

**Table:**

	*x*	*y*	*z*	*U*_iso_*/*U*_eq_	
N5	0.6736 (3)	0.84317 (19)	0.28889 (17)	0.0224 (4)	
C6	0.6810 (3)	0.8500 (2)	0.41148 (19)	0.0188 (4)	
N1	0.8708 (3)	1.2651 (2)	0.74897 (19)	0.0281 (4)	
H1A	0.9098	1.3503	0.8183	0.034*	
N2	0.6143 (3)	0.70917 (18)	0.41274 (16)	0.0192 (3)	
N4	0.5984 (3)	0.69178 (19)	0.21085 (16)	0.0224 (4)	
C3	0.7487 (3)	0.9955 (2)	0.52968 (19)	0.0183 (4)	
C5	0.8008 (4)	1.1310 (3)	0.7624 (2)	0.0301 (5)	
H5	0.7952	1.1308	0.8452	0.036*	
N3	0.5635 (3)	0.61292 (18)	0.28545 (16)	0.0197 (3)	
C2	0.8224 (3)	1.1378 (2)	0.5204 (2)	0.0243 (4)	
H2	0.8309	1.1419	0.4391	0.029*	
C4	0.7372 (4)	0.9933 (2)	0.6525 (2)	0.0253 (4)	
H4	0.6868	0.8994	0.6605	0.030*	
C1	0.8826 (4)	1.2725 (2)	0.6330 (2)	0.0282 (5)	
H1	0.9312	1.3682	0.6280	0.034*	
Co1A	0.41776 (4)	0.36866 (3)	0.20745 (2)	0.01763 (9)	
Cl2	0.08900 (9)	0.38823 (6)	0.08079 (5)	0.02643 (13)	
Cl1	0.73776 (8)	0.35040 (6)	0.33499 (5)	0.02677 (13)	
O1W	0.5652 (3)	0.33958 (19)	0.04986 (14)	0.0285 (3)	
H1WA	0.7173	0.3570	0.0656	0.043*	
H1WB	0.5254	0.3583	−0.0197	0.043*	
O2W	0.2585 (3)	0.37067 (19)	0.35990 (15)	0.0296 (3)	
H2WA	0.3037	0.3809	0.4362	0.044*	
H2WB	0.1266	0.3867	0.3566	0.044*	
O3W	0.2553 (3)	0.12646 (16)	0.12299 (15)	0.0266 (3)	
H3WA	0.1036	0.0973	0.1231	0.040*	
H3WB	0.2462	0.0857	0.0400	0.040*	
O4W	0.1925 (3)	0.96192 (19)	0.85528 (16)	0.0363 (4)	
H4WA	0.2306	1.0107	0.8093	0.054*	
H4WB	0.2283	0.8803	0.8140	0.054*	

### Atomic displacement parameters (Å^2^)

**Table:**

	*U*^11^	*U*^22^	*U*^33^	*U*^12^	*U*^13^	*U*^23^
N5	0.0273 (9)	0.0172 (8)	0.0214 (8)	0.0062 (7)	0.0062 (7)	0.0075 (7)
C6	0.0169 (9)	0.0168 (9)	0.0209 (9)	0.0050 (7)	0.0041 (7)	0.0066 (7)
N1	0.0271 (9)	0.0175 (8)	0.0273 (9)	0.0062 (7)	0.0015 (7)	−0.0020 (7)
N2	0.0221 (8)	0.0155 (7)	0.0161 (8)	0.0052 (6)	0.0026 (6)	0.0040 (6)
N4	0.0280 (9)	0.0182 (8)	0.0191 (8)	0.0063 (7)	0.0049 (7)	0.0071 (7)
C3	0.0140 (8)	0.0161 (9)	0.0216 (9)	0.0056 (7)	0.0001 (7)	0.0053 (7)
C5	0.0356 (12)	0.0251 (11)	0.0225 (10)	0.0083 (9)	0.0043 (9)	0.0045 (9)
N3	0.0235 (8)	0.0161 (8)	0.0173 (8)	0.0058 (7)	0.0045 (7)	0.0054 (6)
C2	0.0254 (10)	0.0200 (10)	0.0282 (11)	0.0077 (8)	0.0066 (8)	0.0107 (8)
C4	0.0319 (11)	0.0189 (9)	0.0231 (10)	0.0082 (8)	0.0042 (8)	0.0075 (8)
C1	0.0247 (10)	0.0163 (9)	0.0405 (13)	0.0064 (8)	0.0065 (9)	0.0093 (9)
Co1A	0.02082 (15)	0.01475 (14)	0.01550 (15)	0.00521 (11)	0.00386 (11)	0.00507 (11)
Cl2	0.0265 (3)	0.0249 (3)	0.0237 (3)	0.0102 (2)	−0.0001 (2)	0.0064 (2)
Cl1	0.0260 (3)	0.0286 (3)	0.0274 (3)	0.0105 (2)	0.0033 (2)	0.0135 (2)
O1W	0.0259 (8)	0.0419 (9)	0.0191 (7)	0.0127 (7)	0.0067 (6)	0.0131 (7)
O2W	0.0331 (9)	0.0381 (9)	0.0230 (8)	0.0158 (7)	0.0124 (7)	0.0140 (7)
O3W	0.0318 (8)	0.0187 (7)	0.0218 (7)	0.0030 (6)	0.0043 (6)	0.0050 (6)
O4W	0.0518 (11)	0.0262 (8)	0.0300 (9)	0.0106 (8)	0.0174 (8)	0.0104 (7)

### Geometric parameters (Å, °)

**Table:**

N5—N4	1.337 (2)	C4—H4	0.93
N5—C6	1.340 (3)	C1—H1	0.93
C6—N2	1.337 (2)	Co1A—O1W	2.0738 (16)
C6—C3	1.467 (3)	Co1A—O2W	2.0933 (16)
N1—C1	1.332 (3)	Co1A—O3W	2.1071 (17)
N1—C5	1.339 (3)	Co1A—Cl1	2.4568 (9)
N1—H1A	0.86	Co1A—Cl2	2.5041 (9)
N2—N3	1.335 (2)	O1W—H1WA	0.94
N4—N3	1.323 (2)	O1W—H1WB	0.88
C3—C4	1.389 (3)	O2W—H2WA	0.83
C3—C2	1.393 (3)	O2W—H2WB	0.91
C5—C4	1.377 (3)	O3W—H3WA	0.94
C5—H5	0.93	O3W—H3WB	0.86
N3—Co1A	2.1153 (18)	O4W—H4WA	0.83
C2—C1	1.379 (3)	O4W—H4WB	0.88
C2—H2	0.93		
			
N4—N5—C6	104.78 (16)	C2—C1—H1	120.1
N2—C6—N5	112.14 (17)	O1W—Co1A—O2W	173.47 (6)
N2—C6—C3	124.23 (18)	O1W—Co1A—O3W	87.50 (7)
N5—C6—C3	123.61 (17)	O2W—Co1A—O3W	86.15 (7)
C1—N1—C5	122.93 (19)	O1W—Co1A—N3	92.57 (7)
C1—N1—H1A	118.5	O2W—Co1A—N3	93.86 (7)
C5—N1—H1A	118.5	O3W—Co1A—N3	176.39 (6)
N3—N2—C6	103.82 (16)	O1W—Co1A—Cl1	89.28 (5)
N3—N4—N5	108.68 (16)	O2W—Co1A—Cl1	89.42 (5)
C4—C3—C2	118.98 (19)	O3W—Co1A—Cl1	92.27 (6)
C4—C3—C6	120.36 (18)	N3—Co1A—Cl1	91.34 (6)
C2—C3—C6	120.65 (19)	O1W—Co1A—Cl2	91.67 (5)
N1—C5—C4	119.4 (2)	O2W—Co1A—Cl2	89.64 (5)
N1—C5—H5	120.3	O3W—Co1A—Cl2	87.85 (6)
C4—C5—H5	120.3	N3—Co1A—Cl2	88.54 (6)
N4—N3—N2	110.58 (15)	Cl1—Co1A—Cl2	179.04 (2)
N4—N3—Co1A	123.34 (12)	Co1A—O1W—H1WA	118.8
N2—N3—Co1A	125.83 (13)	Co1A—O1W—H1WB	125.5
C1—C2—C3	119.3 (2)	H1WA—O1W—H1WB	108.6
C1—C2—H2	120.4	Co1A—O2W—H2WA	131.9
C3—C2—H2	120.4	Co1A—O2W—H2WB	120.0
C5—C4—C3	119.6 (2)	H2WA—O2W—H2WB	106.3
C5—C4—H4	120.2	Co1A—O3W—H3WA	112.7
C3—C4—H4	120.2	Co1A—O3W—H3WB	112.6
N1—C1—C2	119.8 (2)	H3WA—O3W—H3WB	100.6
N1—C1—H1	120.1	H4WA—O4W—H4WB	98.3

### Hydrogen-bond geometry (Å, °)

**Table:**

*D*—H···*A*	*D*—H	H···*A*	*D*···*A*	*D*—H···*A*
O3W—H3WA···O4W^i^	0.95	1.92	2.858 (3)	173
O3W—H3WB···O4W^ii^	0.86	1.91	2.761 (2)	170
O1W—H1WB···N4^iii^	0.88	2.01	2.848 (2)	157
O2W—H2WA···N2^iv^	0.83	2.24	2.999 (2)	153
O4W—H4WA···N5^v^	0.83	2.11	2.935 (3)	173
N1—H1A···Cl2^v^	0.86	2.41	3.180 (2)	149
O1W—H1WA···Cl2^vi^	0.94	2.32	3.254 (2)	174
O2W—H2WB···Cl1^vii^	0.91	2.42	3.300 (2)	163
O4W—H4WB···Cl1^iv^	0.88	2.38	3.249 (2)	168

Symmetry codes: (i) −*x*, −*y*+1, −*z*+1; (ii) *x*, *y*−1, *z*−1; (iii) −*x*+1, −*y*+1, −*z*; (iv) −*x*+1, −*y*+1, −*z*+1; (v) −*x*+1, −*y*+2, −*z*+1; (vi) *x*+1, *y*, *z*; (vii) *x*−1, *y*, *z*.
